# Letter to the Editor From Richmond et al: “Sleep Duration and Visceral Adipose Tissue: Linear and Nonlinear Mendelian Randomization Analyses”

**DOI:** 10.1210/clinem/dgad598

**Published:** 2023-10-12

**Authors:** Rebecca C Richmond, Fergus W Hamilton, George Davey Smith

**Affiliations:** MRC Integrative Epidemiology Unit, University of Bristol, Bristol, BS8 2BN, UK; Population Health Sciences, Bristol Medical School, University of Bristol, Bristol, BS8 2BN, UK; MRC Integrative Epidemiology Unit, University of Bristol, Bristol, BS8 2BN, UK; Department of Infection Science, North Bristol NHS Trust, Bristol, BS10 5NB, UK; MRC Integrative Epidemiology Unit, University of Bristol, Bristol, BS8 2BN, UK; Population Health Sciences, Bristol Medical School, University of Bristol, Bristol, BS8 2BN, UK

Numerous observational studies have identified nonlinear relationships between sleep duration and cardiometabolic risk, many of which highlight a U-shaped relationship with elevated risk among both short (<7 hours) and long (>8 hours) sleepers (1). Nonetheless, previous studies have typically relied on self-reported sleep duration and residual confounding remains problematic. In an attempt to overcome these issues and re-evaluate the relationship between sleep duration and visceral adiposity tissue, Yu et al. performed linear and nonlinear Mendelian randomization (MR) in the UK Biobank (n = 396 858), using 77 single nucleotide polymorphisms (SNPs) associated with sleep duration (2) as instrumental variables.

The authors found evidence for nonlinearity between genetically predicted sleep duration and visceral adipose tissue (VAT), whereby short sleep duration (≤6 hours) increased VAT but with little evidence for an effect of long sleep duration (≥9 hours) on VAT. This L- (rather than U-) shaped relationship is intriguing in suggesting that sleep restriction adversely affects fat distribution, while those who sleep longer than the recommended 7 to 8 hours are not at risk of elevated VAT mass.

While the details given regarding the nonlinear approach used to reach this conclusion are limited, the authors refer to use of the “Nonlinear MR package” and piecewise linear method, which is a variation of the residual (or instrumental variable–free) method (3). This approach divides the population into strata of the (instrumental variable–free) exposure and estimates a localized average causal effect (LACE) in each stratum. Stratum-specific estimates are then combined to generate the shape of the exposure-outcome relationship. Using this method, the authors observed that an L-shaped trend better reflected the relationship between sleep duration and predicted VAT than a linear trend.

We have expressed concern about the use of the residual method for assessing nonlinear causal relationships (4). It relies on an assumption that the genetic effect on the exposure is constant in the population. If this effect is unequal between strata in the nonlinear analysis, this can lead to bias in the within-strata estimates. A more recently developed nonparametric approach, known as the “doubly ranked” stratification method (5), is less sensitive to the constant genetic effect assumption and also allows this assumption to be assessed. However, it is plausible that there are differential degrees of selection into the strata using both approaches, which can produce biased estimates.

Additional sensitivity analyses are recommended to test the robustness of the nonlinear MR methods, including assessing nonlinear trends with different numbers of genetically predicted exposure strata, investigating the distribution of potential confounders within each stratum, and performing negative control analysis. For the latter, nonlinear MR can be performed in relation to traits which are not plausible outcomes, such as age and biological sex. We have recently evaluated both residual and doubly ranked methods using negative controls and identified bias in estimates obtained from both approaches (6). The negative control approach does not reliably identify the source of bias but can reveal that bias is likely to have distorted estimates.

Within this setting, we have conducted a negative control analysis using the 77 SNPs associated with sleep duration and both the residual and doubly ranked methods. We were unable to perform fine stratification of the exposure since hours of sleep duration was recorded in integers and so within small strata there was limited variation in the exposure. Using 3 strata (as performed in the main analysis) and both methods, we found evidence for predicted causal effects of sleep duration on both age and sex, with heterogeneity in estimates between strata and departure from linearity (https://github.com/gushamilton/sleep_non_linear). Analyses were run without covariates but results were similar with the addition of sex (for analysis on age), age (for analysis of sex), UK Biobank recruitment centre, and the first 5 genetic principal components. No such effects were observed in conventional MR analysis of sleep duration on age (Beta 0.10 [95% CI −0.19, 0.40] *P* = .46) and sex (OR 0.94 [95% CI 0.88, 1.01] *P* = .09). As indicated above, findings from the negative control analysis imply that there is bias in the nonlinear MR, which may represent stratum-specific selection bias or bias inherent in the methods. Furthermore, since stratification into as few as 3 strata causes substantial heterogeneity, the stability and goodness of fit of the overall model seems questionable.

The demonstrable bias in the nonlinear MR analysis—which is in agreement with that which has been shown (4, 6) and acknowledged (7) in other settings—is the major issue we would like to raise. However, while the authors should be commended on the extensiveness of the analysis conducted, including numerous sensitivity analyses to assess violation of the instrumental variable assumptions, some other issues should be highlighted.

Since the genome-wide association study used to identify the sleep duration SNPs was conducted in the same population (8), the use of internal weights is not recommended due to bias induced by Winner's curse, whereby genetic effects tend to be exaggerated in the sample in which they are discovered.

Adjusting for phenotypic “confounders” found to be associated with the genetic risk score for sleep duration is not an appropriate method for assessing the “independence” assumption. If these traits lie on the causal pathway, and thus are illegitimate control variables, adjustment would remove real effects and may lead to collider bias, by inducing a spurious association between the genetic instrument and other confounding factors. Alternatively, if these traits are true confounders of the genetic risk score and VAT, adjustment would leave residual confounding due to measurement error.

While the authors have conducted a series of pleiotropy-robust methods (including MR Egger, weighted median, MR-PRESSO and Radial MR), these approaches do not directly account for correlated pleiotropy, whereby the genetic instrument for the exposure is associated with a heritable confounder (eg, psychological trauma) which influences both exposure (sleep duration) and outcome (visceral fat accumulation). Correlated pleiotropy is a particular threat to MR studies which instrument complex behavioral exposures such as sleep duration, and approaches to detect and account for it should be investigated (9).

Finally, the reporting of the nonlinear MR analysis is limited and coefficients for the sleep duration effect on VAT within the strata have not been presented. This makes the quality and reliability of the results difficult to evaluate. We recommend that the STROBE-MR be closely followed and the checklist be completed to accompany any manuscript which conducts Mendelian randomization analysis (10).

## Abbreviations

MR: Mendelian randomization; SNP, single nucleotide polymorphism; VAT, visceral adipose tissue
